# m-EASIX (better than EASIX) predicts severe CAR T-cell toxicities, worse overall survival, and discriminates cytokine release syndrome from sepsis

**DOI:** 10.3389/fimmu.2025.1664788

**Published:** 2026-01-07

**Authors:** Ana Belén Moreno-Castaño, Sara Fernández, Helena Brillembourg, Gontzal Iraola, David F Moreno, Helena Ventosa-Capell, Julia Martinez-Sanchez, Blanca De Moner, Patricia Molina, Alex Ramos, Marta Palomo, Pilar Gómez-Ramírez, Valentín Ortiz-Maldonado, Nuria Martínez-Cibrián, Julio Delgado, Aina Oliver-Caldés, Carlos Fernández de Larrea, María Queralt Salas, Álvaro Urbano, Adrián Téllez, José María Nicolás, Manel Juan, Azucena González-Navarro, Olaf Penack, Gines Escolar, Enric Carreras, Francesc Fernández-Avilés, Pedro Castro, Maribel Diaz-Ricart

**Affiliations:** 1 Hemostasis and Erythropathology Laboratory, Hematopathology, Pathology Department, Centre Diagnòstic Biomèdic (CDB), Clínic Barcelona, Barcelona, Spain; 2 Institut d’Investigacions Biomèdiques August Pi i Sunyer (IDIBAPS), Barcelona, Spain; 3 Facultat de Medicina i Ciències de la Salut, Universitat de Barcelona, Barcelona, Spain; 4 Medical Intensive Care Unit, Clínic Barcelona, Barcelona, Spain; 5 Josep Carreras Leukaemia Research Institute (Campus Clinic), Barcelona, Spain; 6 Hematology External Quality Assessment Laboratory, Centre de Diagnòstic Biomèdic, Clínic Barcelona, Barcelona, Spain; 7 Hematology Department, Institut del Càncer i Malalties de la Sang (ICAMS), IDIBAPS, Hospital Clínic de Barcelona, Barcelona, Spain; 8 Immunology Department, Centre de Diagnòstic Biomèdic, Clínic Barcelona, Barcelona, Spain; 9 Charité Hospital, Berlin, Germany

**Keywords:** biomarkers, CAR T-cells, EASIX, endotheliopathy, m-EASIX, sepsis, toxicities

## Abstract

**Background:**

Cytokine Release Syndrome (CRS) and Immune Effector Cell-Associated Neurotoxicity Syndrome (ICANS) are life-threatening complications that often arise after CAR T-cell immunotherapy. Endothelial dysfunction is believed to play a central role in their development, leading to the interest in biomarker-based tools for diagnosis and differentiating these toxicities from sepsis. This study aimed to evaluate the Endothelial Activation Stress Index (EASIX) and its modified version (m-EASIX, which replaces creatinine with C-reactive protein [CRP] (mg/dL)) as early predictors of severe CRS and ICANS, as well as tools to distinguish CRS from sepsis.

**Methods:**

One hundred and nineteen patients treated with CAR T-cell therapy for CD19-positive hematologic malignancies (n=94) or multiple myeloma (n=23) were included. EASIX and m-EASIX scores were measured at various time points: before CAR T-cell infusion, 24–48 hours post-infusion, at CRS or ICANS onset, and after treatment for each toxicity. A comparator group of 129 sepsis patients, including 86 with hematologic malignancies, was also analyzed.

**Results:**

Both EASIX and m-EASIX correlated with biomarkers of endotheliopathy, with m-EASIX showing stronger predictive power for severe toxicities and ICU admission. Higher EASIX and m-EASIX values at early time points were associated with worse overall survival (OS). Furthermore, m-EASIX accurately distinguished CRS from sepsis at symptom onset.

**Conclusions:**

m-EASIX is a practical and accessible tool for the early prediction of severe CAR T-cell-related toxicities, risk stratification, and differential diagnosis from sepsis, offering potential to guide clinical decision-making and early intervention.

## Introduction

Chimeric antigen receptor (CAR)-T cell immunotherapy has become an effective treatment option for patients with relapsed or refractory oncological and hematological malignancies. However, treatment-related toxicities can be life-threatening and may require management in the intensive care unit (ICU) (1). The most common toxicities are cytokine release syndrome (CRS) and immune effector-cell-associated neurotoxicity syndrome (ICANS) (2, 3), which typically occur shortly after the CAR-T infusion and are pathophysiologically characterized by hyperinflammation (4, 5), capillary leak (6), and, in severe cases, coagulopathy (7).

Growing evidence suggests that endotheliopathy, triggered by a systemic inflammatory cascade, plays a central role in the development of both CRS and ICANS. Several biomarkers reflecting endothelial activation and damage, such as angiopoietin-2 (Ang-2), soluble vascular cell adhesion molecule 1 (sVCAM-1), and von Willebrand factor antigen (VWF: Ag), have been proposed as potential indicators and even predictors of these complications (6–11). However, these markers are not routinely assessed in clinical laboratories, and their implementation in guiding clinical decision-making remains limited.

Recently, the Endothelial Activation Stress Index (EASIX), calculated as lactate dehydrogenase (LDH, U/L) × creatinine (mg/dL) ÷ platelet count (10^9^ cells/L), has emerged as a useful and accessible surrogate marker of endothelial damage. This index can be easily derived from routine blood tests. Initially validated as a predictor of complications and mortality following allogeneic hematopoietic cell transplantation (allo-HCT) (12–17), EASIX has also been investigated as a predictor of CAR-T cell therapy-related toxicities (18–22).

Modifications of the EASIX formula have been proposed to improve its predictive performance. The modified EASIX (m-EASIX), which replaces creatine with C-reactive protein (CRP, mg/dL), has shown value in predicting severe toxicities in both adult (23) and pediatric (22) CAR-T recipients. The addition of CRP and ferritin to pre-lymphodepletion EASIX calculations enhances the predictive value of the score for severe CAR-T cell–related toxicities. Additionally, an EASIX variant incorporating interleukin-10 (IL-10) has demonstrated predictive ability for bleeding events following anti-CD19 CAR-T infusion (24).

To date, the correlation between EASIX and specific endotheliopathy biomarkers in the CAR-T setting has been scarcely explored (18), and its role in predicting sepsis has only been investigated in allo-HCT recipients (25, 26). Nevertheless, the correlations between m-EASIX and endotheliopathy biomarkers, as well as the performance of either score in differentiating CRS from sepsis, have not been investigated.

This study aimed to explore the correlation between EASIX, m-EASIX and circulating biomarkers of endotheliopathy, hemostatic imbalance and innate-immune activation in a cohort of patients treated with different CAR-T products. Additionally, we investigated the utility of these scores as early predictors of severe toxicities, their potential role in early mortality risk stratification, and their ability to differentiate between CRS and sepsis.

## Methods

### Patient population and sample/data collection

We prospectively included adult patients with relapsed/refractory hematologic malignancies who were admitted to our center to receive CAR-T cell immunotherapy between June 2018 and October 2024. Our approach was a prospective, observational cohort study. Eligible patients received one of the following CAR-T-cell products available during the recruitment period: varnimcabtagene autoleucel, axicabtagen ciloleucel, lisocabtagene maraleucel, brexucabtagene autoleucel (all targeting CD19+ malignancies), cesnicabtagene autoleucel (ARI0002h, an academic BCMA-CAR T-cell product for multiple myeloma); and ARI0007 (an academic CD7-CAR T-cell product administered to one patient with acute myeloblastic leukemia). Prior to CAR T-cell administration, all patients received lymphodepleting chemotherapy with fludarabine and cyclophosphamide, as specified by each product. Under our non-randomized approach CAR-T products were allocated based on clinical criteria, product availability, and disease indication.

Clinical data were extracted from electronic medical records. They included the following variables: age, sex, significant comorbidities, underlying hematologic malignancy, previous treatments and related complications, disease status at admission, diagnosis of sepsis during hospitalization, and occurrence, timing, and severity grading of CAR-T cell-related toxicities. Information on the management of toxicities, including response to therapy, need for additional interventions, and intensive care unit (ICU) admission, was also recorded.

Patients with active infections due to Human Immunodeficiency Virus, hepatitis C virus, or hepatitis B virus were excluded.

Blood samples were collected at predefined time points throughout the immunotherapy course: before CAR-T cell infusion (time point A); 24–48h post-infusion (B); at the time of CRS suspicion if compatible signs and symptoms were present (C); 72–96h after CRS treatment (D); at the time of ICANS suspicion if compatible signs and symptoms were present (E); and 72–96h after ICANS treatment (F). If patients did not present any toxicity, a control sample was collected 96h after CAR T-cell infusion (G). Plasma was obtained in 3.2% citrated tubes and separated by centrifugation at 3000 g within 4 hours after extraction, aliquoted, and stored at −40 °C until use. Serum was obtained in serum-separating tubes, centrifuged at 3000g, and processed on the day as part of routine examinations.

In a previously published study (11), 62 of the included CAR T-cell patients underwent biomarker analyses performed in plasma samples. Markers of endothelial dysfunction, measured by ELISA, were the soluble vascular cell adhesion molecule 1 (sVCAM-1) (Sigma-Aldrich, USA); soluble TNF receptor 1 (sTNFRI) (Biomatik, Delaware, USA); soluble suppression of tumorigenesis-2 factor (sST2); thrombomodulin (TM), and angiopoietin-2 (Ang-2) (R&D Systems, Minnesota, USA). Biomarkers of innate immunity and complement activation included Neutrophil extracellular traps (NETs), using the Quant-iT PicoGreen dsDNA Assay Kit (Invitrogen, Thermo Fisher, Massachusetts, USA) by fluorimetry (Fluoroskan Ascent FL; Thermolab Systems, Massachusetts, USA); the soluble terminal fraction of the complement system membrane attack complex (sC5b-9), determined by ELISA (Quidel, California, USA). The hemostasis and fibrinolysis imbalance biomarkers measured included plasma levels of circulating von Willebrand Factor antigen (VWF: Ag) (vWF Ag, Siemens Healthineers, Germany) and α2-Antiplasmin activity (α2-AP), by Berichrom α2-Antiplasmin Kit, in the Atellica 360 COAG coagulometer (Siemens Healthineers, Germany); ADAMTS-13 activity (A13), by fluorescence resonance energy transfer (FRET-VWF73); plasminogen activator inhibitor-1 antigen (PAI-1 Ag), by ELISA (Imubind, Toronto, Canada). Each assay was performed in one run, including all samples in the same batch, and carried out as blinded operator assays. The levels of the different biomarkers were correlated with EASIX and m-EASIX scores.

Laboratory parameters used to calculate EASIX (LDH (U/L), creatinine (mg/dL), platelet count (10^9^ cells/L)), and m-EASIX (replacing creatinine with CRP (mg/dL)) were determined within the routine tests in serum samples (Advia 2400, Siemens Healthineers, Erlangen, Germany) and retrospectively collected at the same time points. When multiple values were available on the same day, the highest values for LDH, creatinine, and CRP, and the lowest platelet count were used. As platelet transfusion are systematically registered in the electronic medical records, the pre-transfusional value was selected). Log2-transformed values of EASIX and m-EASIX were also calculated for subsequent analyses.

When a positive microbiological culture occurred in the hospitalization period after CAR T-cell infusion and was deemed sufficient to explain the clinical presentation (e.g., fever, hypotension, or hypoxia), the episode was classified as sepsis rather than CRS. Although overlap between syndromes is possible, this dichotomization was applied for analytical purposes. Patients who experienced both a toxicity episode and, later, a clearly separated sepsis episode during the same hospitalization, were included in both the toxicity and sepsis subanalyses. For each analysis, the parameters used to calculate the scores corresponded to the onset of the respective syndrome.

Treatment-related mortality (TRM) was defined as death occurring within 100 days after CAR T-cell infusion. Response to CAR-T cell treatment was assessed one month after therapy in all patients.

Finally, data from CAR-T cell recipients were compared with an independent historical cohort of 129 ICU patients (86 of whom had hematologic malignancies) admitted between 2014 and 2024 for sepsis or septic shock, following sepsis-3–2016 criteria (27). In these patients, the scores EASIX and m-EASIX were calculated retrospectively only at the time of their admission to the ICU.

### Statistical analysis

Descriptive data are presented as frequencies and percentages for categorical variables, and as mean ± standard deviation (SD) or median ± interquartile range (IQR) for continuous variables, depending on the distribution. Normality was assessed using appropriate tests before selecting parametric or non-parametric methods. Univariate analyses were performed accordingly.

To evaluate the temporal evolution of EASIX and m-EASIX scores within patients across different time points during immunotherapy, paired comparisons were conducted using the Wilcoxon signed-rank test.

The association of EASIX, m-EASIX, and other clinical or laboratory variables with relevant outcomes, namely the development of severe toxicities, treatment-related mortality, and ICU admission, was assessed using logistic regression models. Binary logistic regression was used for dichotomous outcomes, while linear regression was applied for continuous dependent variables. Significant predictors were reported with corresponding odds ratios (ORs) and 95% confidence intervals (CIs). To assess the individual contribution of each component included in the EASIX and m-EASIX formulas, separate univariate logistic regressions were performed, never including the index and its component parameters in the same model to avoid multicollinearity. The Variance Inflation Factor (VIF) was calculated for CRP in models that included m-EASIX. VIFs below 5 were considered acceptable.

Correlations between EASIX, m-EASIX and the different biomarkers of endotheliopathy were analyzed with Pearson coefficients.

Receiver Operating Characteristic (ROC) curves were generated, and areas under the curve (AUCs) were calculated to evaluate the discriminatory capacity of EASIX and m-EASIX for different outcomes, including severe toxicity, ICU admission, and sepsis. Bootstrap corrections of AUCs based on 100 replicates were applied for each analysis. Brier scores, Hosmer-Lemeshow test and deviations between predicted and observed probabilities were used to complement ROC analysis of the main predictive models.

For survival analysis, overall survival (OS) was defined as time between CAR-T infusion and death for any cause. Univariate Cox proportional hazards analyses were first performed for all candidate variables impacting overall survival (OS). Those variables reaching statistical significance were included in multivariable Cox models, for which the proportional hazards assumption was verified using Schoenfeld residuals. Kaplan–Meier (KM) curves and log-rank tests were used to compare survival between categorical subgroups, excluding groups with very small sample sizes. Moreover, KM curves were also applied after patients were dichotomized into “high” and “low” EASIX or m-EASIX groups based on optimal cutoff points determined using log-rank statistics with the maxstat package in R. Statistical significance was defined as a two-tailed p-value <0.05.

All analyses and graphics were performed using R version 4.3.2. The following R packages were used: dplyr, survival, maxstat, car, ggplot2, pROC, boot, rmda, and rms. Statistical code is available from the corresponding author upon reasonable request. No formal power calculation was conducted; the sample size was determined based on all eligible patients treated at our center during the study period. Missing data were handled using the pairwise deletion method. No data imputation was applied. Outliers were retained unless linked to laboratory error.

### Ethics

This study was approved by the ethics committee of Hospital Clinic Barcelona (Registry HCB/2021/0608). All patients provided informed consent prior to their inclusion in the study.

De-identified data supporting the findings of this study are available from the corresponding author upon reasonable request. No public data repository was used due to privacy restrictions.

## Results

### Patients’ characteristics

A total of 119 patients who received CAR T-cell therapy were included in the analysis. Baseline clinical characteristics at the time of inclusion, the specific CAR-T construct administered, and the incidence of associated toxicities are summarized in **Table 1**.

**Table 1:** Characteristics of the cohort of CAR T-cell patients included.

Variables	n (Total=119)
Age (years)	55 (19–78)
Gender (female)	46 (38)
Hematologic malignancy	
CD19 +	94 (78.9)
Acute lymphoblastic leukemia	29 (24.4)
DLBC (and aggressive transformations from indolent lymphomas)	43 (36.1)
Mantle cell lymphoma	7 (6)
Indolent B cell lymphoproliferative disorders	15 (12.6)
Multiple myeloma/Plasma cell dyscrasias	23 (19.3)
Other^a^	2 (1.6)
Construct received	
Varnimcabtagene autoleucel	51 (43)
Cesnicabtagene autoleucel	23 (19.3)
Axicabtagene Ciloleucel	40 (33.6)
Lisocabtagene maraleucel	3 (2.5)
Others^b^	2 (1.6)
Re-infusion (yes)	11 (9.2)
Fractionated infusion (yes)	68 (57)
Disease status before CAR-T	
Complete response	18 (15.1)
Partial response	9 (7.6)
Very good partial response	3 (2.5)
Therapeutic failure	81 (68.1)
Not available	8 (6.7)
≥2 previous therapeutic lines	84 (70.6)
Previous auto-HCT (yes)	40 (33.6)
Previous allo-HCT (yes)	28 (23.5)
Previous treatment with Inotuzumab-Ozogamicin (yes)	19 (16)
CRS	76 (64)
Grade ≥ 2 or persistent CRS grade 1	37 (31)
Grade ≥ 3	4 (3.3)
Onset of CRS (days after infusion)	3 (5)
ICANS; n (%)	23 (19.3)
Grade ≥ 3	8 (6.7)
Onset of ICANS (days after infusion)	6 (4)
CRS + ICANS	22 (18.5)
ICU admission	26 (22)

Categorical variables are expressed as frequency (percentage); quantitative variables are expressed as median (Interquartile range).

a n=1 Hodgkin lymphoma transformed to DLBCL and n=1 acute myeloblastic leukemia.

b n=1 brexucabtagene autoleucel, n=1 (ARI0007).

auto-HCT, autologous hematopoietic cell transplantation; allo-HCT, allogeneic cell transplantation; CRS, cytokine release syndrome; DLBC, Diffuse large B cell lymphoma ICANS, Immune effector cell-associated neurotoxicity syndrome; IQR, interquartile range; ICU, intensive care unit.

**Table 2:** Baseline characteristics of patients developing CRS in the CAR T-cell cohort and septic patients (from the CAR T-cell cohort and historical sepsis cohort) included for the comparison of EASIX and m-EASIX scores in CRS vs. Sepsis.

	CAR T patients with CRS and/or confirmed sepsis n=78	Septic patients (historical cohort) n=129	p
Age, years	55 (20)	65 (23)	0.00067
Positive microbiological culture	19 (24)	107 (83)	2.2 ·10^-16^
Clinical criteria for sepsis	10 (13)	129 (100)	1.2 ·10^-8^
^a^Comorbidities that can impact on endothelial function	26 (33)	86 (66)	6.2 ·10^-6^
ICU admission	26 (33)	129 (100)	2.2 ·10^-16^
SOFA	5 (3)	8 (5)	0.00023
APACHE-II	20 (11)	16 (10)	0.13
Transfusion within 1 week before sample collection	24 (31)	56 (43)	0.096

Categorical variables are expressed as frequency (percentage); quantitative variables are expressed as median (Interquartile range). P value was obtained after analyzing differences between variables in both groups using the Wilcoxon test for continuous variables and the Chi-square test for categorical variables.

Clinical criteria of sepsis: organ dysfunction with a change in SOFA score >2 and infection with a clinically significant positive result in a microbiological culture.

a Comorbidities: included in this section are diseases or conditions that might cause potentially endothelial damage and affect baseline values of the EASIX or m-EASIX indices. In this variable are considered cardiovascular risk factors (heart disease, hypertension, diabetes, dyslipidemia), chronic kidney disease, and chronic inflammatory disease or infection (i.e., autoimmune disease, HIV infection).

ICU, intensive care unit; SOFA, Sequential Organ Failure Assessment score; APACHE-II, Acute Physiology and Chronic Health Evaluation II. Transfusion includes platelets or red blood cells, as both have been demonstrated to potentially alter parameters that conform to the scores EASIX and m-EASIX.

CRS of any grade was observed in 76 patients, while 23 patients developed ICANS. All patients who experienced ICANS had received the full dose of the CAR-T product in a single aliquot, and in 21 of the 23 cases, the construct administered was axicabtagene ciloleucel. Confirmed sepsis, defined as having clinical criteria of sepsis (organ dysfunction with a change in SOFA score ≥2 and infection with a clinically significant positive result in a microbiological culture) was diagnosed in 10 patients in the CAR T-cell cohort.

### Correlation between EASIX and m-EASIX scores and biomarkers of endotheliopathy and related pathways

**Figure 1:**
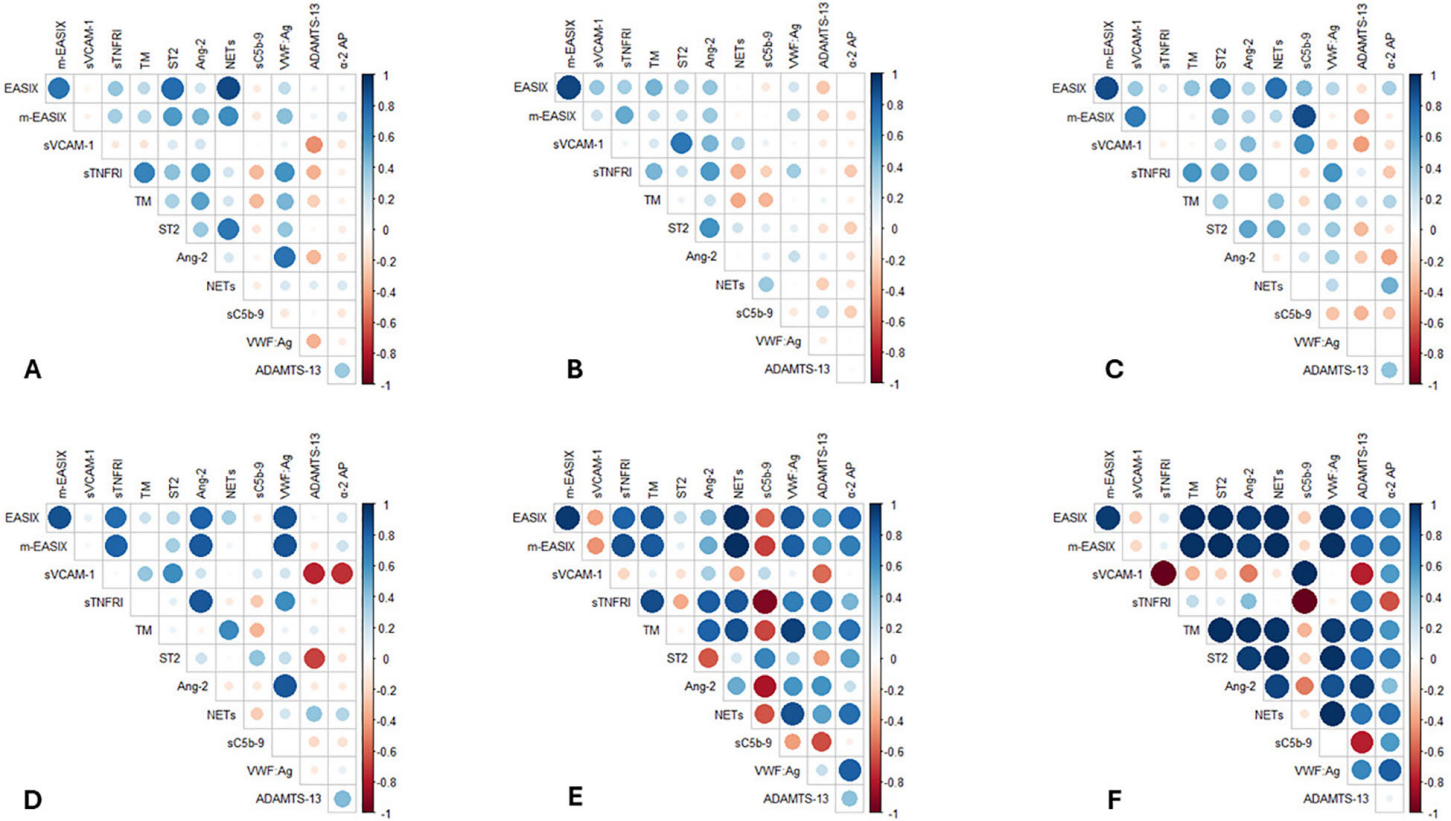
Correlation matrices between EASIX and m-EASIX scores and biomarkers of endotheliopathy and hemostasis imbalance, fibrinolysis and innate-immune activation at the different time points. Blue circles represent positive correlations, and red circles represent negative correlations. Only significant correlations are shown (p < 0.05, as determined by Pearson analysis); otherwise, the intersection box is blank. The intensity of the color and the size of the circle are proportional to the correlation coefficient. **(A)** Pre-infusion (baseline) time point. **(B)** Early post-infusional time point. **(C)** CRS onset time point, if presented. **(D)** Time point after CRS treatment with immunomodulatory agents, if required. **(E)** ICANS onset time point, if presented. **(F)** Time point after ICANS treatment with corticosteroids, if required.

We assessed the correlation between EASIX and m-EASIX scores and circulating biomarkers of endotheliopathy, hemostasis imbalance, fibrinolysis, and innate immune activation across multiple time points during the immunotherapy course (**Figure 1**). Overall, both EASIX and m-EASIX presented a positive correlation with most biomarkers, except for ADAMTS-13 and sC5b9, which demonstrated negative correlations with the scores at certain time points. Importantly, the relationship between the scores and biomarkers varied over time, reflecting the dynamic nature of endothelial and immune activation during CAR T-cell therapy. The most relevant changes were observed at the onset of ICANS, where both scores displayed a strong negative correlation with sVCAM-1 and sC5b-9 (**Figure 1E**). These inverse correlations were partially attenuated following ICANS-directed treatment (**Figure 1F**).

### Baseline EASIX and m-EASIX as surrogates of endotheliopathy depend on preexisting clinical conditions

Baseline EASIX and m-EASIX values varied according to patients past medical history. Patients with lymphoid neoplasms (n= 95) exhibited significantly higher EASIX and m-EASIX scores compared to those with plasma-cell dyscrasias (n=23) (mean and IQR: EASIX 1.39 ± 1.29 vs. 0.8 ± 0.56, respectively, p=0.008); m-EASIX 1.59 ± 3.99 vs. 0.63 ± 1.75, respectively, p=0.026).

Patients with only a partial response or progressive disease before CAR T-cell infusion (n=90) had significantly higher m-EASIX scores compared to those who were in complete remission or very good partial response (n=21) (1.84 ± 4.25 vs. 0.41 ± 1.48, respectively; p=0.00032). No significant differences were observed in baseline EASIX between groups.

Similarly, patients who had previously undergone autologous hematopoietic cell transplantation (auto-HCT) (n=40) exhibited lower m-EASIX values than those who had not (n=79) (1.12 ± 1.9 vs. 1.65 ± 5.67, p=0.032), whereas patients with a history of acute graft-versus-host disease (GVHD) following allogeneic HCT (n=13) had significantly lower EASIX scores than those without GVHD (n=12) (0.99 ± 1.16 vs. 2.43 ± 3.12, p= 0.026). This was not observed for m-EASIX. No differences in these scores were observed between patients with (n=3) or without previous chronic GVHD, although the small sample size limits interpretation.

There were no significant differences in baseline EASIX or m-EASIX scores with respect to the number of previous treatment lines or previous exposure to inotuzumab-ozogamicin (n=19) in patients with ALL.

### Kinetics of EASIX and m-EASIX during the immunotherapy

No significant differences were observed in EASIX or m-EASIX values between baseline (time point A) and the immediate post-infusion period (time point B), in the overall cohort.

However, among patients who developed CRS, a significant increase in m-EASIX values was observed from time point A to time point B (1.61 ± 3.34 vs. 2.56 ± 6.1, respectively; p = 0.003247). Also, both EASIX and m-EASIX significantly increased at the time of CRS onset (point C) compared to the post-infusion values (point B) (EASIX: 1.82 ± 2.13 vs. 1.38 ± 1.45; p=4.1·10^-6^; m-EASIX: 11.83 ± 22.64, p=1.14 ·10^-9^.

**Figure 2:**
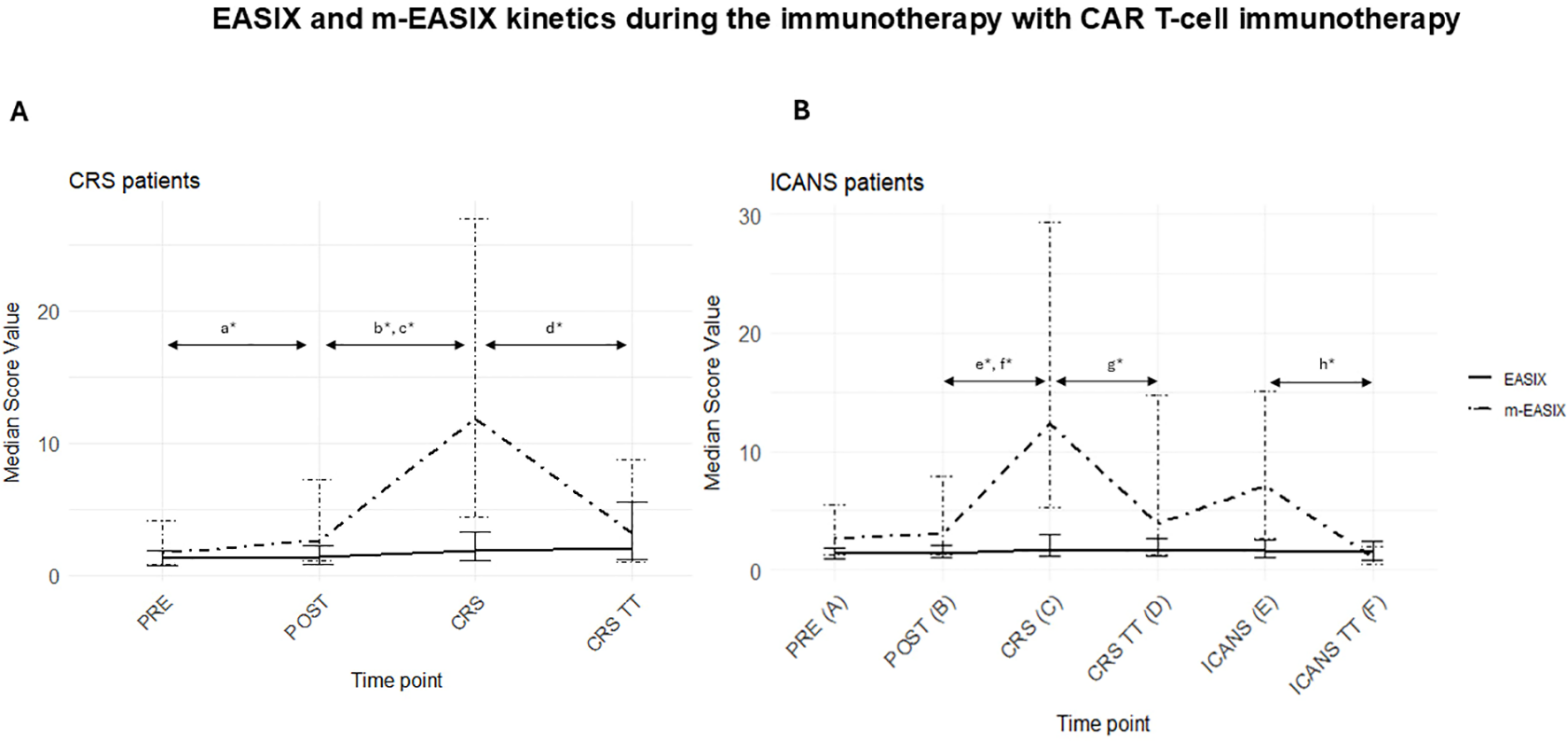
**(A)** Kinetics of EASIX and m-EASIX during the immunotherapy with CAR T-cells in patients who presented CRS (n=76). Dispersion is expressed in the Interquartile Range (IQR). Statistical signficancy, determined by the Wilcoxon signed-rank test, is marked with the symbol *. a* Median of m-EASIX at time point A (pre-infusion) vs. at time point B (early post-infusion), p=0,0032 b* Median of EASIX at time point B (early post-infusion) vs. at time point C (CRS onset), p=4.1·10^-6^. c* Median of m-EASIX at time point B (early post infusion) vs. time point C (CRS onset), p=1.14·10^-9^. d* Median of m-EASIX at point C (CRS onset) vs. D (post CRS treatment), p=5.4·10^-7^. **(B)** Kinetics of EASIX and m-EASIX during the immunotherapy with CAR-T cells in patients who presented ICANS (n=23). Notably, all ICANS patients, except one, had presented with CRS before undergoing ICANS. Dispersion is expressed in the Interquartile Range (IQR). e* Median of EASIX at time point B (early post infusion) vs. at time point C (CRS onset), p=0.0121. f* Median of m-EASIX at time point B (early post infusion) vs. time point C (CRS onset), p=0.0006815. g* Median of m-EASIX at time point C (CRS onset) vs. D (post specific CRS treatment), p=0.02157. h*Median of m-EASIX at point E (ICANS onset) vs. Point F (after corticosteroid treatment), p=0.0003847.

Following the administration of CRS-specific therapy, a significant decrease in m-EASIX, but not in EASIX, was observed (m-EASIX: 11.83 ± 22.64 at point C vs. 3.16 ± 7.73 at point D; p), p=5.4 ·10^-7^; EASIX: 1.82 ± 2.13 at point C vs. 2.02 ± 4.33 at point D; p=0.256) (**Figure 2A**). In patients who developed ICANS, neither EASIX nor m-EASIX showed significant changes at ICANS onset (point E) compared to either post-infusion (point B) or CRS onset values (point C). Nevertheless, m-EASIX, and again not EASIX, significantly decreased following corticosteroid treatment (m-EASIX: 7.1 ± 12.5 at point E vs. 1.03 ± 1.43 at point F, p=0.00038; EASIX: 1.57 ± 1.42 at point E vs. 1.47 ± 1.54 at point F, p=0.527) (**Figure 2B****).**

Patients who did not develop CRS or ICANS did not exhibit significant changes in EASIX or m-EASIX values throughout immunotherapy (median values of EASIX were 1.06 and m-EASIX were 0.87 at all time points). Different sub-analyses were applied to assess the kinetics of the scores during immunotherapy with the CAR T-cell constructs with more casuistry. The same tendency was observed with all CAR T-cell products, as in the entire cohort, although not all changes were significant due to the limited sample size in some subgroups (**Supplementary Figures S1**-**S4**).

### Performance of EASIX and m-EASIX as early predictors of severe CAR T-cell toxicity and ICU admission

The predictive value of EASIX and m-EASIX was analyzed at baseline (time point A) and after infusion (time point B). For the prediction of severe toxicities, 77 patients were included, and for the ICU admission subanalysis, 118 patients were analyzed, representing those with sufficient data available for each analysis.

At baseline, patients who developed severe (grade ≥3) toxicities had significantly higher m-EASIX values compared to those who did not (4.65 ± 6.3 vs. 1.51 ± 3.1, p=0.0254). In contrast, no significant difference was observed in EASIX values (1.4 ± 1.5 vs.1.3 ± 1.1, p=0.56).

**Figure 3:**
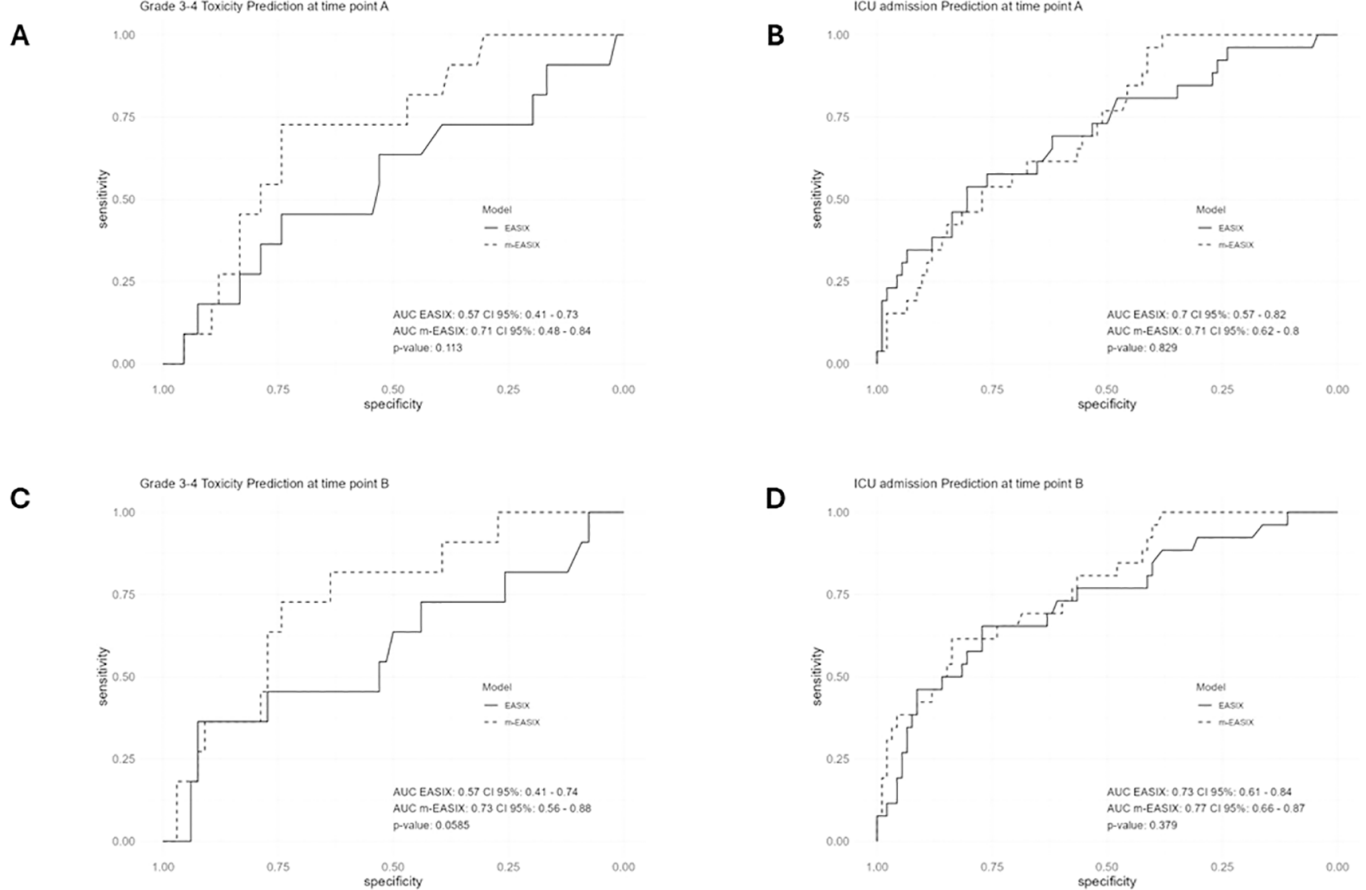
ROC analysis for the performance of EASIX and m-EASIX for early prediction of any Grade 3–4 Toxicity (at time points A and B in Panels A and C, respectively) and for need for Intensive Care Unit admission (at time point A and B in panels B and D, respectively). Data corrected by bootstrap after 100 replicates. The respective index’s threshold, calculated with Youden optimal cutoff, with the best sensitivity and specificity for the respective outcome are as follows: For panel **(A)** EASIX threshold 1.83, sensitivity 45%, specificity 74%; m-EASIX threshold 3.69, sensitivity 73%, specificity 74%. For panel **(B)** EASIX threshold 1.83, sensitivity 54%, specificity 80%; m-EASIX threshold 0.69, sensitivity 100%, specificity 38%. For panel **(C)** EASIX threshold 4.29, sensitivity 36%, specificity 92%; m-EASIX threshold 5.94, sensitivity 73%, specificity 74%. For panel **(D)** EASIX threshold 1.7, sensitivity 65%, specificity 77%; m-EASIX threshold 5.94, sensitivity 62%, specificity 84%.

ROC curve analysis, corrected by bootstrap based on 100 replicates, showed that baseline m-EASIX outperformed EASIX and their log_2_-transformed counterparts in predicting severe toxicities, with AUCs of 71% for m-EASIX (CI 95% 0.48-0.84), 68% for log_2_ m-EASIX (CI 95% 0.46-0.82), 57% for EASIX (CI 95% 0.41-0.73), and 57% for log_2_ EASIX (CI 95% 0.43-0.73) (**Figure 3A**). A baseline m-EASIX value > 3.68 had a sensitivity of 73% and specificity of 74% for predicting grade ≥3 CRS or ICANS (**Figure 3A**). For the prediction of ICU admission, both scores showed similar performance at time point A: AUC of m-EASIX 71% (CI 95% 0.62-0.80), AUC of EASIX 70% (CI 95% 0.57-0.82), AUC of log_2_ m-EASIX 65% (CI 95% 0.41-0.77), and AUC of log_2_ EASIX 0.70 (CI 95% 0.54-0.81). The m-EASIX threshold >0.69 yielded a 100% sensitivity and 38% specificity for this outcome (**Figure 3B**).

Univariate logistic regression at baseline revealed that CRP was the only individual score component significantly related to the development of severe toxicities (OR 1.3, 95% CI 1.01-1.6, p = 0.042). In contrast, a low platelet count was the only variable significantly associated with ICU admission (OR: 0.99, 95% CI: 0.98-0.99, p = 0.03).

At the post-infusion time point (B), m-EASIX remained significantly higher in patients who later developed grade ≥3 toxicities compared to those who did not (7.4 ± 13.4 vs. 2.3 ± 5.4, p = 0.01, while EASIX values did not differ significantly (1.4 ± 3.4 vs.1.4 ± 1.4, p=0.38). Again, and after bootstrap analysis correction, m-EASIX demonstrated superior predictive accuracy for severe toxicity compared to the other scores at point B: AUC of m-EASIX 73% (CI 95% 0.56-0.88), AUC of EASIX 57% (CI 95% 0.41-0.74), AUC of log_2_ m-EASIX 70% (CI 95% 0.51-0.87), and AUC of log_2_ EASIX 60% (CI 95% 0.45-0.77) (**Figure 3C**). After conducting performance assessments, the predictive model based on m-EASIX at time point B for ≥3 grade toxicities displayed no global miscalibration (Brier score 0.122 and Hosmer-Lemeshow test p=0.18). In addition, the average deviation error was 0.11 while the maximum error was 0.65, suggesting that the average differences of the predicted and observed probabilities were small, though a local miscalibration was observed due to the small sample size.

Moreover, m-EASIX also showed the best predictive power for ICU admission a time point B: AUC of m-EASIX 77% (CI 95% 0.66-0.87), AUC of EASIX 73% (CI: 0.61-0.84), AUC of log_2_ m-EASIX 73% (CI 95% 0.53-0.85), and AUC of log_2_ EASIX 72% (CI 95% 0.60-0.83). A m-EASIX cutoff >5.94 provided a 62% sensitivity and 84% specificity for the prediction of need for ICU admission at this time point (**Figure 3D**). After applying model refinement analyses, we did not observe global miscalibration for m-EASIX at time point B for the prediction of ICU admission (Brier score 0.14, Hosmer-Lemeshow test p=0.28). The average calibration deviation was 0.08 and the maximum deviation was 0.29, therefore local miscalibration was smaller for the ICU model compared to the ≥3 grade toxicities model.

At time point B, CRP was again the only individual parameter significantly associated with severe toxicity (OR 1.4, 95% CI 1.01-1.8, p = 0.009). In contrast, elevated LDH and CRP levels and low platelet count were independently associated to ICU admission: elevated LDH had an OR of 1.004, (95% CI 1.0002-1.007, p=0.04), elevated CRP had an OR of 1.38 (95% CI 1.13-1.68, p=0.0012), and decreased platelet count had an OR of 0.99 (95% CI 0.98-0.99, p=0.011). In the multivariant regression, only elevated CRP and low platelet count remained independently associated with ICU admission. The Variance Inflation Factor (VIF) was calculated for CRP in models including m-EASIX, yielding values of 1.30 and 1.29 for the prediction of severe toxicity and ICU admission, respectively. These low VIF values indicate mild correlation but fall well below commonly accepted thresholds for problematic multicollinearity (VIF > 5). This finding supports the fact that m-EASIX’s predictive performance is not solely driven by CRP. While the role of CRP alone for the prediction of severe toxicities and need for ICU admission was acceptable (AUC of 0.71 and 0.72 for each outcome, respectively) the values were slightly lower than for m-EASIX, although not significantly.

The performance of the scores at predicting these outcomes at early time points depending on the main groups of CAR T-cell products is shown in **Table 1**. **Supplementary Materials**.

### Performance of EASIX and m-EASIX as early predictors of overall survival, treatment related mortality, early treatment response, and time to relapse

**Figure 4:**
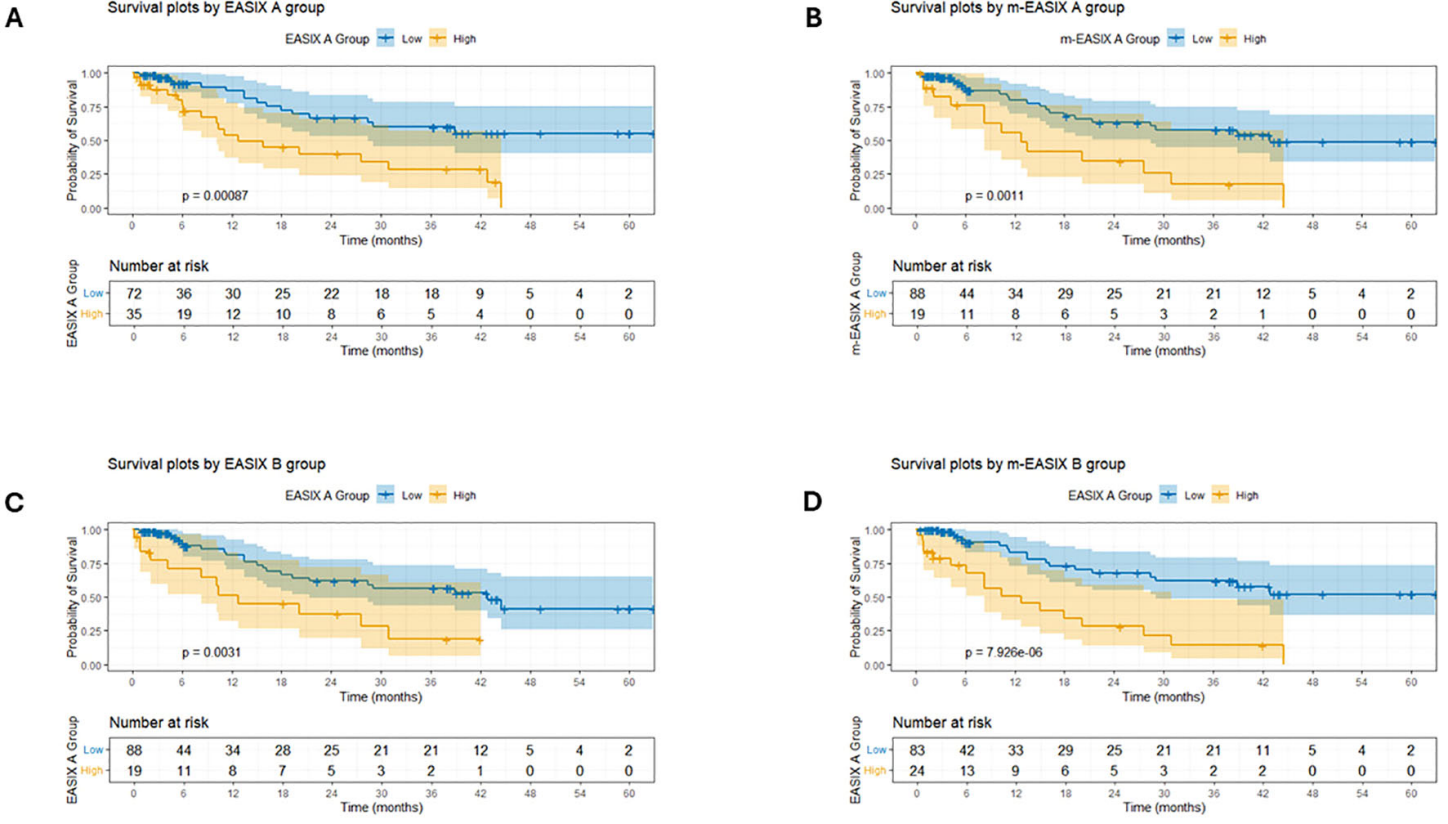
Survival plots in months by EASIX and m-EASIX groups defined by optimal cutoff point (LogRank and Maxstat analysis) at time points A (pre-infusion or baseline) and B (early post-infusion). **(A)** The “High” group includes patients with EASIX values greater than 1.61 at time-point A; the “Low” group includes patients with EASIX values equal to or below this cutoff. **(B)** “High” group includes patients with m-EASIX values >2.84 at time-point A; “Low” group includes patients with m-EASIX values equal or below this cutoff. **(C)** “High” group includes patients with EASIX values >3.08 at time point B; “Low” group includes patients with EASIX values equal or below this cutoff. **(D)** “High” group includes patients with m-EASIX values >8.31 at time point B; “Low” group includes patients with m-EASIX values equal or below this cutoff.

Patients with baseline or post-infusion EASIX or m-EASIX values above the optimal cutoff thresholds had significantly worse overall survival (OS) compared to those with lower scores. This association was evident for both scores, but more significant for m-EASIX at both time points: pre-infusion (**Figures 4A, B**, p = 0.0011 for m-EASIX) and after infusion (**Figures 4C, D**, p= 7.93·10–^6^ for m-EASIX at time point B**).** Moreover, m-EASIX demonstrated exemplary performance as a mortality risk stratifier when subanalyzing patients with lymphoid neoplasms and plasma cell dyscrasias (**Supplementary Figure S5**).

In multivariable Cox proportional hazards analyses, including variables that were significant in univariate models, higher m-EASIX remained an independent predictor of poorer OS (**Table 3**). When probability of survival was assessed according to CAR-T type (only including the three constructs with more casuistry), no significant differences were observed (**Supplementary Figure S6**).

**Table 3:** Multivariable Cox proportional hazards model for overall survival.

Multivariant Cox model: HR with CI95%				
Variable	HR	CI_low	CI_high	p
Age	0.992	0.968	1.017	0.539
CAR T type	0.692	0.496	0.967	0.031
EASIX point A	0.967	0.838	1.115	0.643
m-EASIX point A	1.006	1.001	1.011	0.030
EASIX point B	0.822	0.651	1.037	0.099
m-EASIX point B	1.052	1.016	1.089	0.004

Hazard ratios (HR) with 95% confidence intervals (95% CI) and p-values are shown for each covariate. The model satisfied the proportional hazards assumption (global test p = 0.404).

In contrast, neither EASIX nor m-EASIX demonstrated significant predictive value for treatment-related mortality (TRM), with p-values of 0.31 and 0.57 at baseline, and 0.18 and 0.65 post-infusion, respectively. Similarly, neither scores were significantly associated with early treatment response (assessed at 1 month) (p=0.28 and 0.91 at baseline; p=0.38 and 0.48 post-infusion for EASIX and m-EASIX, respectively), nor with time to relapse among patients who eventually relapsed (p=0.74 and p=0.81 at baseline; p=0.17 and 0.13 post-infusion, for EASIX and m-EASIX, respectively).

### Performance of EASIX and m-EASIX in differentiating CRS from sepsis

**Figure 5:**
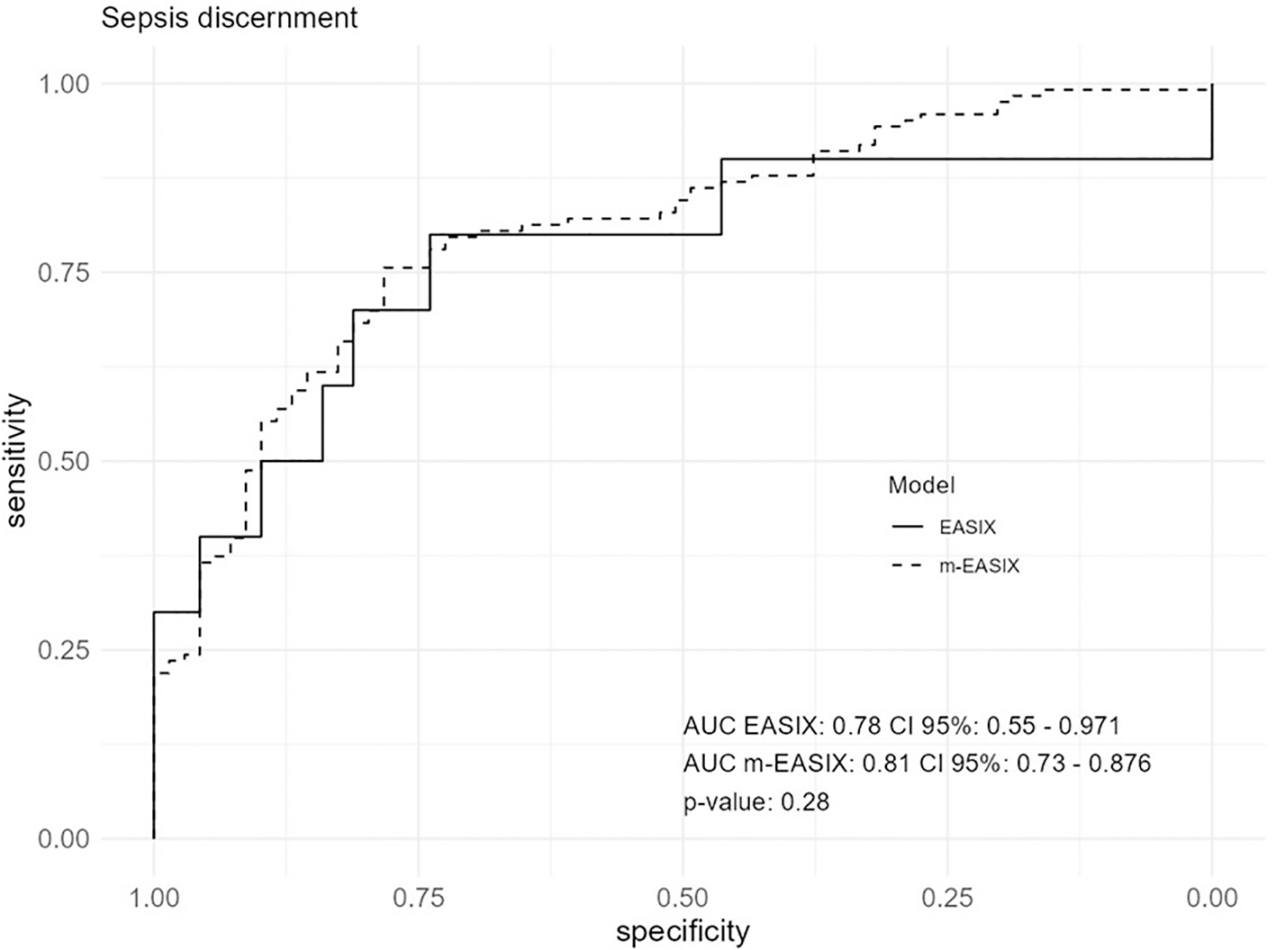
Performance of EASIX and m-EASIX in discriminating Sepsis from CRS at symptoms onset (fever, hypoxia or/and hypotension). The 10 CAR T-cell patients with confirmed sepsis were considered “septic”, in addition to the 129 patients from the historical cohort. Area under the curve (AUC) and Confidence Intervals (CI) corrected by bootstrap after 100 replicates. The respective index’s threshold, calculated with Youden optimal cutoff, with the best sensitivity and specificity for the outcome are as follows: EASIX threshold 2.92, sensitivity 80%, specificity 74%; m-EASIX threshold 26.38, sensitivity 75%, specificity 78%.

ROC analyses were performed to evaluate the diagnostic performance of EASIX and m-EASIX in distinguishing between CRS and sepsis. Patients with CRS were compared with two groups: 1) patients with confirmed sepsis following CAR T-cell infusion (n=10), and 2) a historical cohort of ICU patients with sepsis (n=119), including 86 with hematologic malignancies. The scores were calculated at the onset of symptoms suggestive of either CRS or sepsis (fever, hypoxia, hypotension), or at ICU admission in the historical cohort. In **Table 2** are displayed the baseline characteristics of the patients included in this subanalysis.

m-EASIX outperformed EASIX in discriminating sepsis from CRS, with an AUC of 81% vs. 78%, respectively, although the difference between the scores was not statistically significant (p=0.28) (**Figure 5**).

When the analysis was restricted to severely ill ICU patients (defined as those with an APACHE-II score greater than 12 points), EASIX showed the best performance (AUC 77%), outperforming m-EASIX (AUC 62%), p = 0.0072.

Among the score components, elevated CRP (OR 1.15, 95% CI 1.10-1.21) and elevated creatinine (OR 4.31, 95% CI 1.50-12.40) were independently associated with sepsis diagnosis.

## Discussion

We investigated the role of EASIX and m-EASIX as early predictors of severe toxicities following CAR-T cell immunotherapy, as early mortality risk stratifiers, and as tools for differential diagnosis between CRS and sepsis. Additionally, we assessed the correlation between both scores and specific biomarkers of endotheliopathy, hemostatic imbalance, and innate immune activation. Elevated values of EASIX, and particularly m-EASIX, at early time points were associated with increased risk of severe toxicity, ICU admission, and worse overall survival. Furthermore, both scores proved useful in distinguishing CRS from sepsis at the onset of symptoms.

Endothelial injury is increasingly recognized as a key pathophysiological mechanism underlying complications of cellular therapies. Conditions such as sinusoidal obstructive syndrome and transplant-associated thrombotic microangiopathy after allo-HCT, engraftment syndrome following autologous-HCT, and, more recently, CRS and ICANS after CAR T-cell therapy have all been linked to endothelial activation and dysfunction (12, 13, 16, 18, 23, 28, 29). EASIX, originally proposed as a surrogate marker of endothelial damage, has been correlated with circulating biomarkers of endotheliopathy in various settings, including coronary disease and allo-HCT (29–32). However, data in the context of CAR T-cell therapy remain scarce (18).

In our cohort, EASIX and m-EASIX consistently correlated with most of endothelial and immune biomarkers assessed (including sTNFRI, TM, ST2, Ang-2, NETs, and VWF: Ag). Nevertheless, the strength and direction of these correlations varied depending on the timing and clinical presentation. For example, ADAMTS-13 showed a negative correlation with both scores in the early post-infusion phase and at the onset of CRS, but a strong positive correlation during ICANS and after corticosteroid treatment. This finding may reflect systemic inflammation associated with CAR T-cell expansion, a known inhibitor of ADAMTS-13 synthesis (33–36). In contrast, ICANS may involve a more localized process with lesser systemic inflammatory burden, sparing hepatic ADAMTS-13 production. A similar rationale may explain the observed positive correlation between EASIX/m-EASIX and both sVCAM-1 and sC5b-9 at CRS onset, which reversed at ICANS onset. These results contrast with recent findings (37) and, therefore, further research is warranted to specifically explore the potential differential pathophysiology between ICANS and CRS.

Patients’ medical history influenced baseline EASIX and m-EASIX values. Lower m-EASIX values were observed in patients with previous autologous HCT, consistent with our earlier observation of reduced sVCAM-1 levels in this population (11). This could represent an “exhaustion” phenomenon following sustained endothelial activation and biomarker release (38, 39). Similarly, patients with prior acute GVHD had lower baseline EASIX, possibly reflecting endothelial adaptation or depletion.

Consistent with previous findings from our group, patients with lymphoid malignancies had higher baseline EASIX and m-EASIX scores than those with plasma-cell dyscrasias (11), likely reflecting differences in disease biology or prior treatments. Interestingly, m-EASIX demonstrated a more dynamic trajectory than EASIX in reflecting clinical evolution, particularly during CAR T-related toxicities. This ability could be attributed to the inclusion of CRP in the m-EASIX formula, a biomarker known to independently predict CAR T-cell toxicity (22, 40), and likely a major driver of m-EASIX’s superior predictive value. CRP is an acute-phase reactant released by hepatocytes in response to inflammatory cytokines, such as IL-6, and exerts an inhibitory action on the immune system by suppressing T-cell and dendritic cell differentiation and interactions (41–43). It is therefore plausible that CRP plays a pivotal role in the early diagnosis and prediction of immune-related toxicities associated with cellular therapy, where the secretion of pro-inflammatory cytokines represents the primary pathophysiological trigger. In our study, as in other cohorts treated with cellular therapies (44, 45), CRP alone demonstrated acceptable performance in predicting severe toxicities. However, m-EASIX achieved a superior AUC, supporting the notion that its additional components make a meaningful contribution to the model.

EASIX has been validated in predicting complications after allo-HCT and CAR-T cell therapy, particularly with anti-CD19 products (12–16, 18). In some studies, m-EASIX has also been associated with severe CRS and ICANS in this context (22, 23). Our study not only confirms these findings but also provides a comparative analysis, demonstrating the superiority of m-EASIX in predicting grade 3 or higher CRS/ICANS and ICU admission. In addition, m-EASIX was more strongly associated with poorer OS, at both time points, before and after infusion. Based on the predictive models we have developed, m-EASIX could be helpful for the early identification of patients who may require special attention—such as close monitoring or even preemptive transfer to the ICU, if feasible.

Although the preemptive administration of tocilizumab has been investigated by some research groups, it has not yet been widely adopted in routine clinical practice. The potential role of m-EASIX in guiding specific therapeutic decisions warrants further investigation in clinical trials specifically designed for this purpose.

Among the individual parameters composing EASIX and m-EASIX, CRP and platelet count were the most relevant predictors of toxicity and ICU admission, consistent with prior reports (23). Although platelets play a central role in the scores, their levels in CAR T-cell recipients may reflect multiple factors beyond endothelial dysfunction, such as lymphodepletion-induced aplasia, bone marrow infiltration, infections, and prior therapies. Similarly, LDH elevations may be due to tumor lysis rather than microangiopathic processes. Platelet or red blood cell transfusions may also have an impact on the parameters composing the indexes (46, 47). However, in the present study, transfusion did not show a significant impact on the outcomes, and there were no differences between the CAR T-cell cohort and the septic cohort.

m-EASIX also emerged as the most accurate score for distinguishing between CRS and sepsis. Elevated creatinine was an independent discriminator, underscoring that while both are systemic inflammatory syndromes, sepsis typically involves greater organ dysfunction. Acute kidney injury (AKI) is common in septic ICU patients (43, 48, 49), even among those without severe illness (50, 51). In contrast, AKI is less frequent in CAR T-cell recipients (52, 53) and tends to occur in the setting of severe CRS (54).

This study has several limitations. First, the consecutive inclusion of CAR T-cell recipients resulted in uneven group sizes across different constructs, limiting some subgroup analyses. Second, the retrospective design of the septic patient cohort, along with the baseline differences between comparative groups in terms of underlying disease, comorbidities, and clinical severity before score calculation, as well as the potential changes in sepsis definition and management during the study period, warrants a cautious interpretation of the ROC model for CRS vs. sepsis discrimination. Third, the response to treatment was only assessed one month after immunotherapy and, therefore, longer-term outcomes such as sustained treatment response or relapse were not fully analyzed.

Nevertheless, the study has notable strengths. It includes a large cohort of patients treated with a diverse range of CAR T-cell constructs, beyond anti-CD19 products, thereby increasing the generalizability of our findings to real-world hematologic immunotherapy settings. Furthermore, we conducted comparisons with a well-characterized cohort of sepsis patients previously studied from an endothelial perspective, which is mainly composed of hematologic patients, allowing us to identify novel findings in the differential endothelial profiles of CRS and sepsis, based on their pathophysiology and associated organ dysfunction. Further prospective studies should be proposed to validate these results in larger cohorts of patients, which would also provide a greater number of severe toxicities and sepsis cases to enable comparisons between more homogeneous patient groups.

## Conclusions

EASIX and m-EASIX may serve as complementary surrogate markers of endotheliopathy at different time points during CAR T-cell therapy, as both correlate acceptably with circulating biomarkers of endothelial dysfunction. Although these indices can be influenced by other conditions affecting their components, such as inflammation, tumor proliferation, or renal dysfunction, our results indicate that m-EASIX, overall, represents a feasible and superior tool compared to EASIX for the early prediction of severe toxicities, mortality risk stratification, and differentiation between CRS and sepsis. Nevertheless, validation in larger cohorts will be necessary, ideally allowing stratification by comorbidity groups to assess its independent value better and minimize potential confounding effects.

## Data Availability

The raw data supporting the conclusions of this article will be made available by the authors, without undue reservation.

## Supplementary material

Supplementary Figure 1 Kinetics of EASIX and m-EASIX during the immunotherapy with Varnimcabtagene autoleucel in patients who presented CRS (n=26). Dispersion is expressed in the Interquartile Range (IQR). Statistical significance applying Wilcoxon signed-rank test is marked with the symbol *. a* Median of m-EASIX at time point A (pre infusion) vs. at time point B (early post infusion), p<0.0051. b*Median of m-EASIX at time point B (early post infusion) vs. at time point C (CRS onset), p<0.0024. c* Median of m-EASIX at point C (CRS onset) vs. D (post-specific CRS treatment), p=5.36·10^-7^. There were no patients treated with Varnimcabtagene autoleucel presenting ICANS.

Supplementary Figure 2 Kinetics of EASIX and m-EASIX during the immunotherapy with Axicabtagene Ciloleucel in patients who presented CRS (n=34). Dispersion is expressed in Interquartile Range (IQR). Statistical significance applying Wilcoxon signed-rank test is marked with the symbol *. a* Median of EASIX at time point B (early post-infusion) vs. at time point C (CRS onset), p=0.0072. b*Median of m-EASIX at time point B (early post infusion) vs. at time point C (CRS onset), p=4.8e-05. c* Median of m-EASIX at point C (CRS onset) vs. D (post-specific CRS treatment), p<0.00065.

Supplementary Figure 3 Kinetics of EASIX and m-EASIX during the immunotherapy with Axicabtagene Ciloleucel in patients who presented ICANS (n=21). Dispersion is expressed in the Interquartile Range (IQR). Statistical significance, as determined by the Wilcoxon signed-rank test, is marked with the symbol *. a* Median of EASIX at time point B (early post-infusion) vs. at time point C (CRS onset), p=0.028. b*Median of m-EASIX at time point B (early post infusion) vs. at time point C (CRS onset), p=0.0017. c* Median of m-EASIX at point C (CRS onset) vs. D (post-specific CRS treatment), p=0.049.

Supplementary Figure 4 Kinetics of EASIX and m-EASIX during the immunotherapy with Cesnicabtagene autoleucel in patients who presented CRS (n=13). Dispersion is expressed in Interquartile Range (IQR). Statistical significance applying Wilcoxon signed-rank test is marked with the symbol *. a* Median of m-EASIX at time point A (pre-infusion) vs. at time point B (early post-infusion), p=0,041. b* Median of EASIX at time point B (early post-infusion) vs. at time point C (CRS onset), p=0.00073. c*Median of m-EASIX at time point B (early post infusion) vs. at time point C (CRS onset), p=0.0012.

Supplementary Figure 5 Survival pots by m-EASIX group measured in time point B in CAR T patients with lymphoid diseases and plasma-cell dyscrasias. In panel **(A)**, “High” group includes patients with post infusional m-EASIX values >8.31; “Low” group comprises patients with m-EASIX values equal or below this cutoff. In panel **(B)**, “High” group includes patients with post-infusional m-EASIX values >2.81; “Low” group includes patients with m-EASIX values equal to or below this cutoff.

Supplementary Figure 6 Kaplan-Meier curves showing the probability of survival according to CAR-T type (only including the patients receiving the constructs with more casuistry: Varnimcabtagene autoleucel, Axicabtagene Ciloleucel, Cesnicabtagene autoleucel.

Supplementary Table 1 Subgroup analysis of the performance of the scores EASIX and m-EASIX in predicting ≥ grade 3 toxicities and need for Intensive Care Unit admission at early time points, depending on the main groups of CAR T-cell products. AUC: area under the curve. CI: confidence interval. Corrected by bootstrap based on 100 replicates for each analysis. Correspondence of time points: A: pre-infusional, B: 24-48h post-infusion. ICU: Intensive Care Unit. No patient in Cesnicabtagene autoleucel group presented ≥grade 3–4 toxicities; therefore, this group was not included in this sub-analysis. The other subgroups of CAR T-cell products were not included in this sub-analysis due to their scarce casuistry.
