# Supplementary figures and images for: m-EASIX (better than EASIX) predicts severe CAR T-cell toxicities, worse overall survival, and discriminates cytokine release syndrome from sepsis
Source: Front Immunol. 2026 Jan 7;16:1664788. doi: 10.3389/fimmu.2025.1664788 (PMC12819699; doi:10.3389/fimmu.2025.1664788)

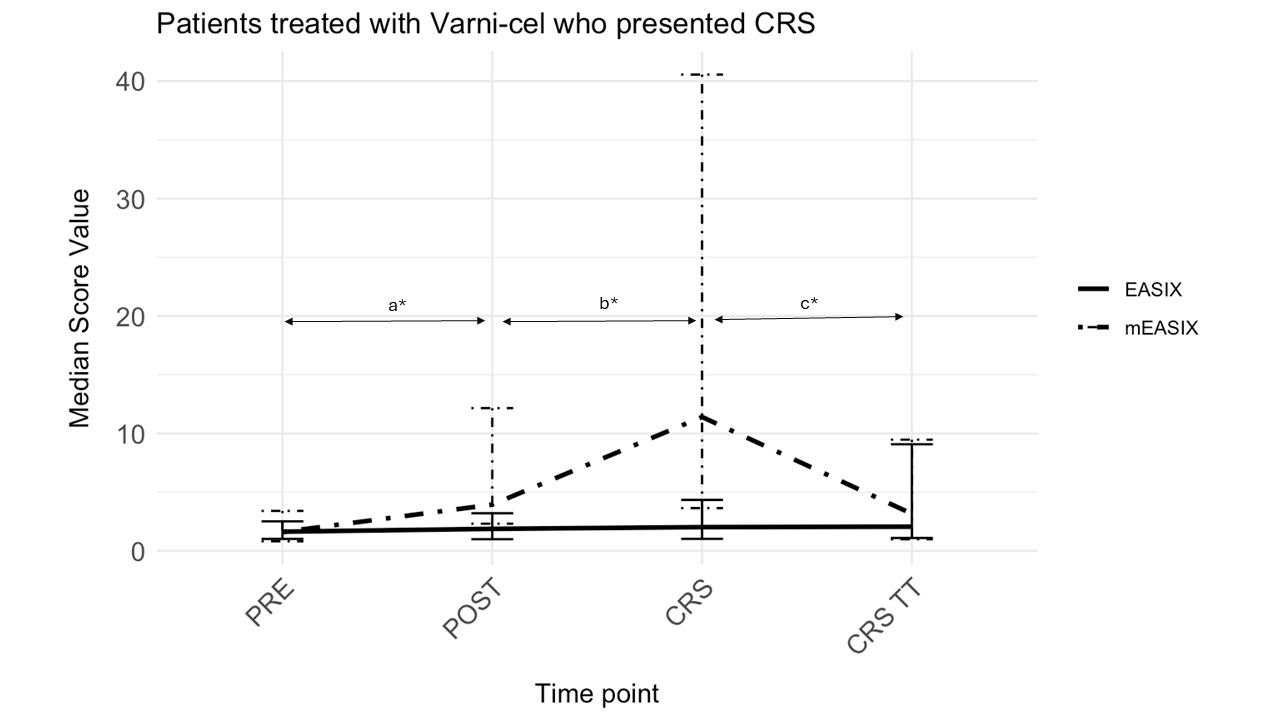

Supplement: Supplementary Figure 1 — Kinetics of EASIX and m-EASIX during the immunotherapy with Varnimcabtagene autoleucel in patients who presented CRS (n=26). Dispersion is expressed in the Interquartile Range (IQR). Statistical significance applying Wilcoxon signed-rank test is marked with the symbol *. a* Median of m-EASIX at time point A (pre infusion) vs. at time point B (early post infusion), p<0.0051. b*Median of m-EASIX at time point B (early post infusion) vs. at time point C (CRS onset), p<0.0024. c* Median of m-EASIX at point C (CRS onset) vs. D (post-specific CRS treatment), p=5.36·10-7. There were no patients treated with Varnimcabtagene autoleucel presenting ICANS. [file Image1.jpeg]

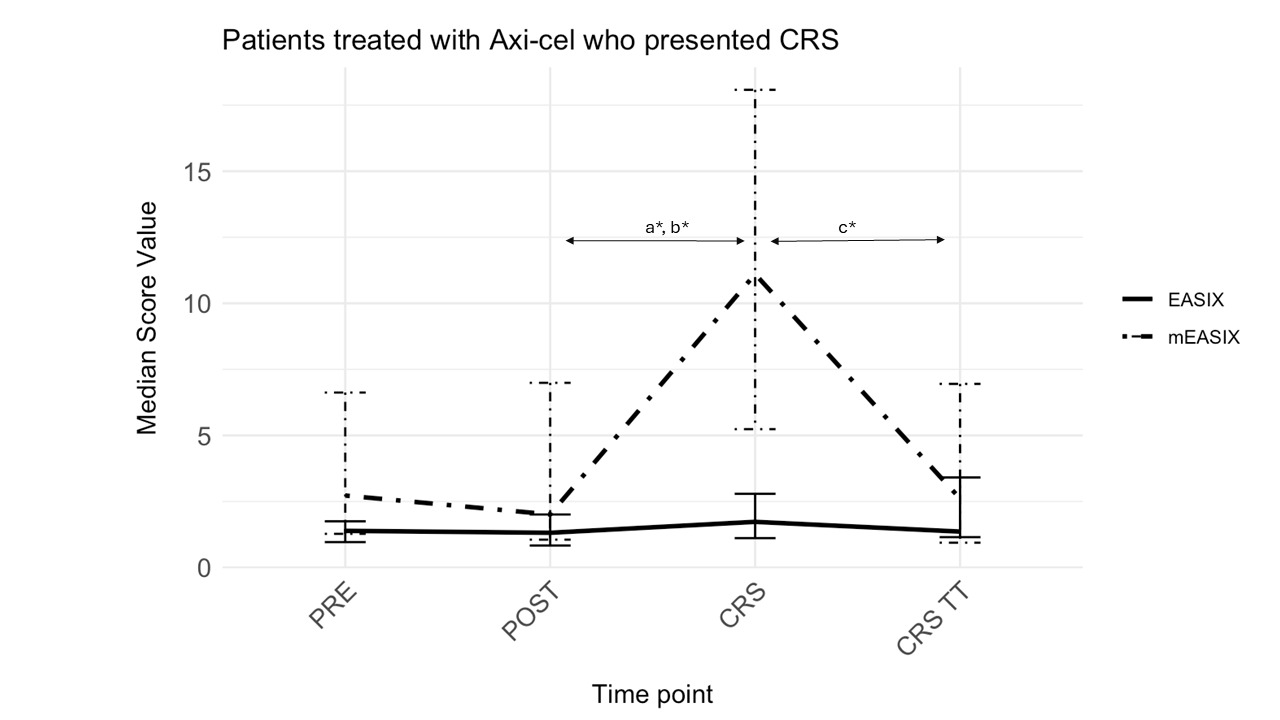

Supplement: Supplementary Figure 2 — Kinetics of EASIX and m-EASIX during the immunotherapy with Axicabtagene Ciloleucel in patients who presented CRS (n=34). Dispersion is expressed in Interquartile Range (IQR). Statistical significance applying Wilcoxon signed-rank test is marked with the symbol *. a* Median of EASIX at time point B (early post-infusion) vs. at time point C (CRS onset), p=0.0072. b*Median of m-EASIX at time point B (early post infusion) vs. at time point C (CRS onset), p=4.8e-05. c* Median of m-EASIX at point C (CRS onset) vs. D (post-specific CRS treatment), p<0.00065. [file Image2.jpeg]

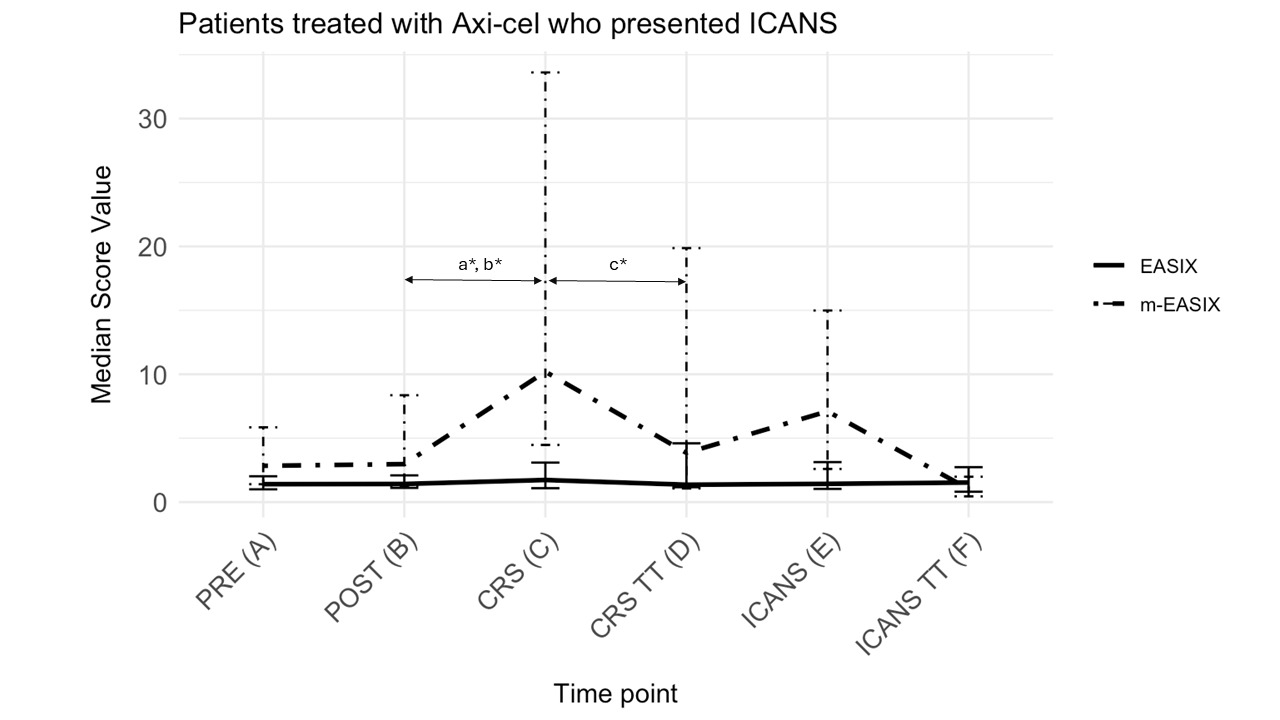

Supplement: Supplementary Figure 3 — Kinetics of EASIX and m-EASIX during the immunotherapy with Axicabtagene Ciloleucel in patients who presented ICANS (n=21). Dispersion is expressed in the Interquartile Range (IQR). Statistical significance, as determined by the Wilcoxon signed-rank test, is marked with the symbol *. a* Median of EASIX at time point B (early post-infusion) vs. at time point C (CRS onset), p=0.028. b*Median of m-EASIX at time point B (early post infusion) vs. at time point C (CRS onset), p=0.0017. c* Median of m-EASIX at point C (CRS onset) vs. D (post-specific CRS treatment), p=0.049. [file Image3.jpeg]

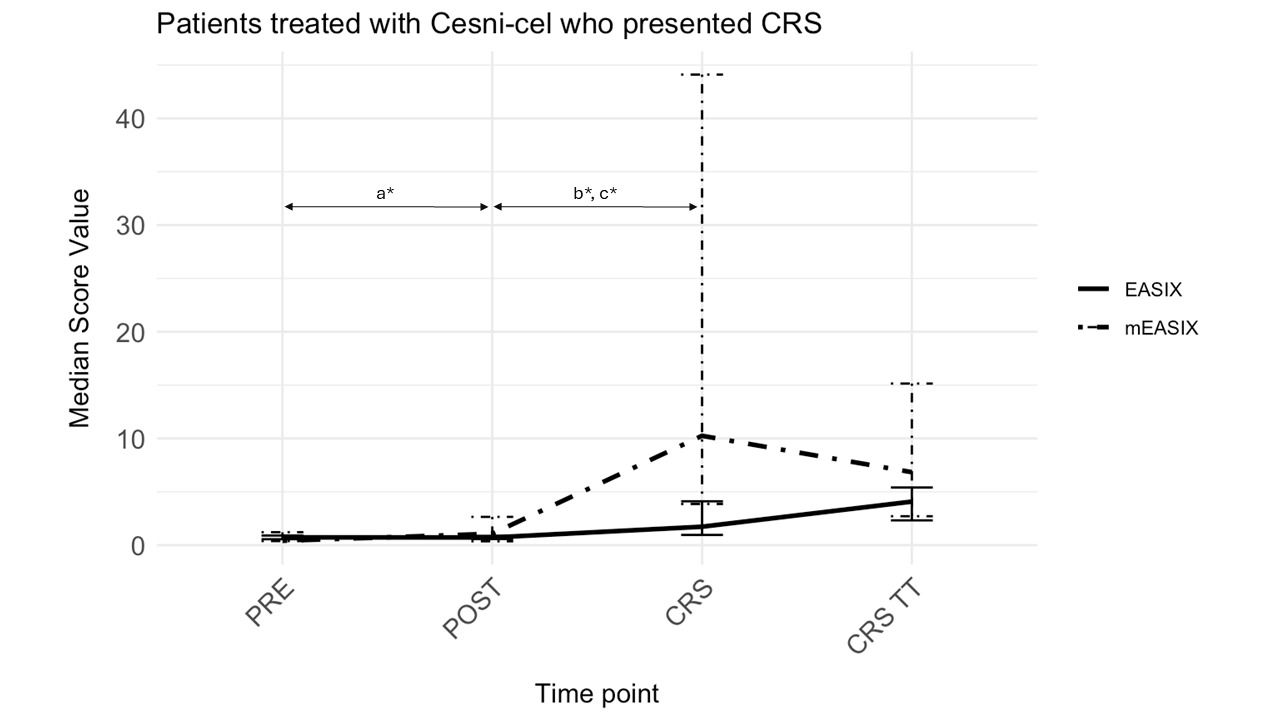

Supplement: Supplementary Figure 4 — Kinetics of EASIX and m-EASIX during the immunotherapy with Cesnicabtagene autoleucel in patients who presented CRS (n=13). Dispersion is expressed in Interquartile Range (IQR). Statistical significance applying Wilcoxon signed-rank test is marked with the symbol *. a* Median of m-EASIX at time point A (pre-infusion) vs. at time point B (early post-infusion), p=0,041. b* Median of EASIX at time point B (early post-infusion) vs. at time point C (CRS onset), p=0.00073. c*Median of m-EASIX at time point B (early post infusion) vs. at time point C (CRS onset), p=0.0012. [file Image4.jpeg]

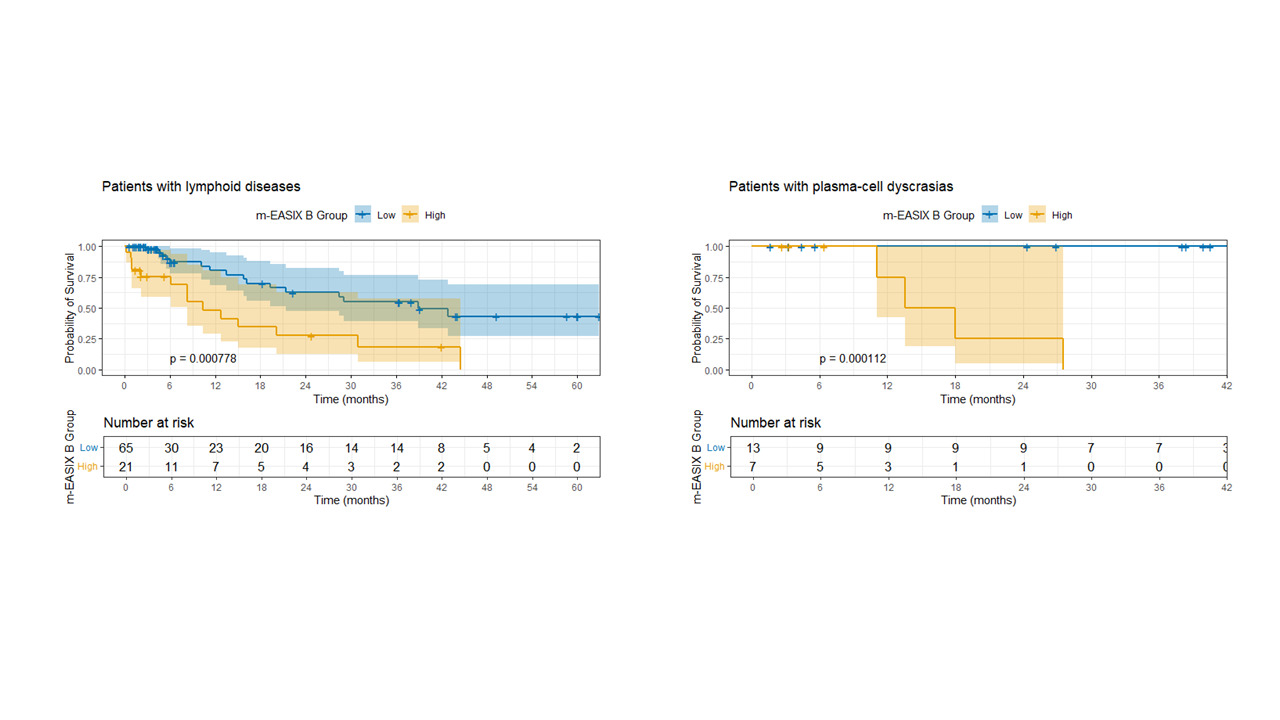

Supplement: Supplementary Figure 5 — Survival pots by m-EASIX group measured in time point B in CAR T patients with lymphoid diseases and plasma-cell dyscrasias. In panel (A), “High” group includes patients with post infusional m-EASIX values >8.31; “Low” group comprises patients with m-EASIX values equal or below this cutoff. In panel (B), “High” group includes patients with post-infusional m-EASIX values >2.81; “Low” group includes patients with m-EASIX values equal to or below this cutoff. [file Image5.jpeg]

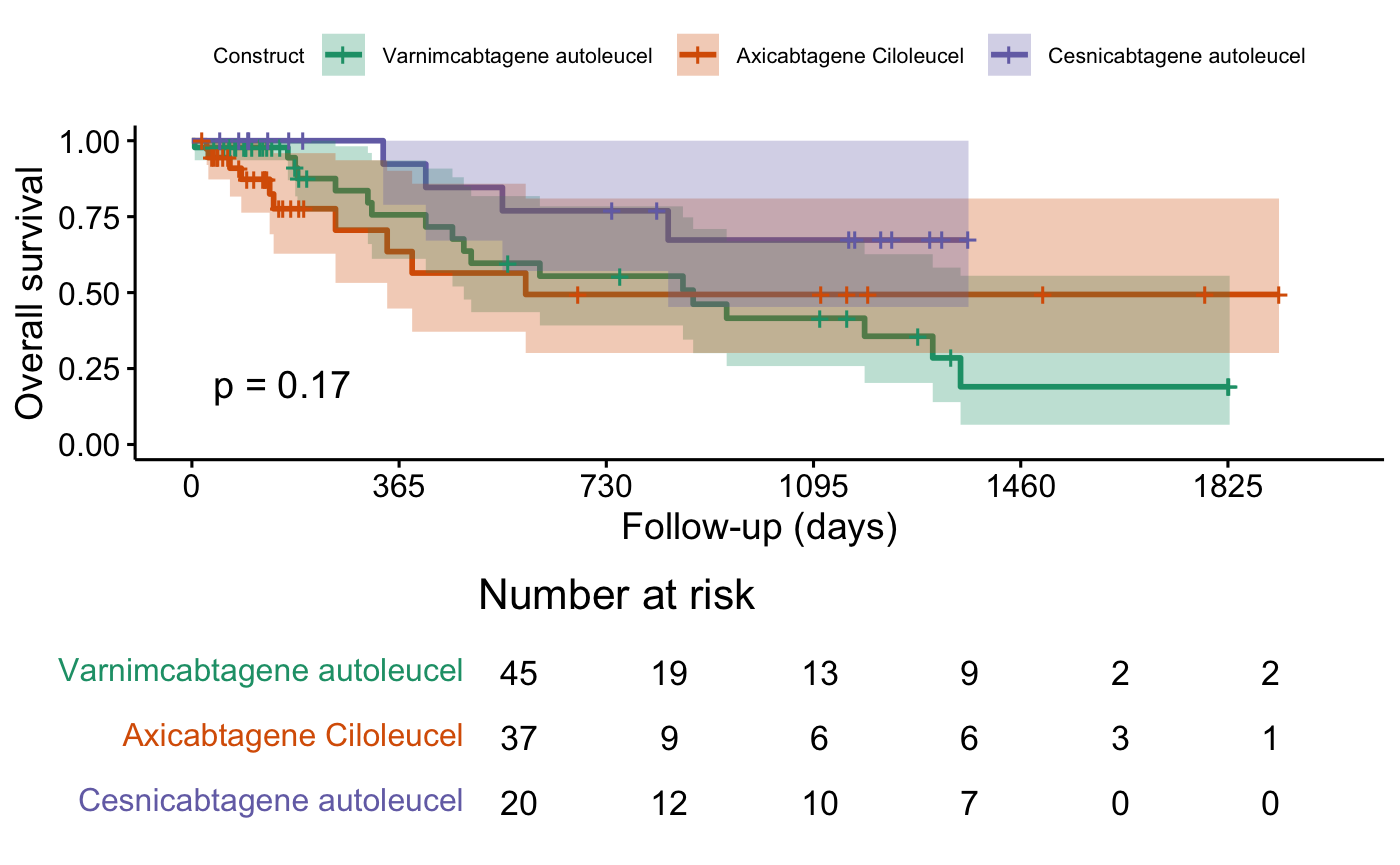

Supplement: Supplementary Figure 6 — Kaplan-Meier curves showing the probability of survival according to CAR-T type (only including the patients receiving the constructs with more casuistry: Varnimcabtagene autoleucel, Axicabtagene Ciloleucel, Cesnicabtagene autoleucel. [file Image6.png]
