# Supplementary material for: m-EASIX (better than EASIX) predicts severe CAR T-cell toxicities, worse overall survival, and discriminates cytokine release syndrome from sepsis
Source: Front Immunol. 2026 Jan 7;16:1664788. doi: 10.3389/fimmu.2025.1664788 (PMC12819699; doi:10.3389/fimmu.2025.1664788)
Supplement: Supplementary Table 1 — Subgroup analysis of the performance of the scores EASIX and m-EASIX in predicting ≥ grade 3 toxicities and need for Intensive Care Unit admission at early time points, depending on the main groups of CAR T-cell products. AUC: area under the curve. CI: confidence interval. Corrected by bootstrap based on 100 replicates for each analysis. Correspondence of time points: A: pre-infusional, B: 24-48h post-infusion. ICU: Intensive Care Unit. No patient in Cesnicabtagene autoleucel group presented ≥grade 3–4 toxicities; therefore, this group was not included in this sub-analysis. The other subgroups of CAR T-cell products were not included in this sub-analysis due to their scarce casuistry. [file Table1.docx]

**Supplemental Materials. Table 1.**

Subgroup analysis of the performance of the scores EASIX and m-EASIX in predicting ≥grade 3-4 toxicities and need for Intensive Care Unit admission at early time points, depending on the main groups of CAR T-cell products.

|  | **AUC** | **CI 95%** |
| --- | --- | --- |
| **Prediction of grade 3-4 Toxicities** | | |
| Varnimcabtagene autoleucel  group | | |
| EASIX at point A | 0.83 | 0.72-0.97 |
| EASIX at point B | 0.87 | 0.75-0.97 |
| m-EASIX at point A | 0.90 | 0.81-0.98 |
| m-EASIX at point B | 0.86 | 0.73-0.96 |
| Axicabtagene Ciloleucel  group | | |
| EASIX at point A | 0.71 | 0.60-0.90 |
| EASIX at point B | 0.70 | 0.58-0.88 |
| m-EASIX at point A | 0.73 | 0.59-0.93 |
| m-EASIX at point B | 0.78 | 0.64-0.95 |
| **Prediction of ICU admission** | | |
| Varnimcabtagene autoleucel  group | | |
| EASIX at point A | 0.74 | 0.64-0.85 |
| EASIX at point B | 0.78 | 0.68-0.92 |
| m-EASIX at point A | 0.74 | 0.62-0.88 |
| m-EASIX at point B | 0.82 | 0.72-0.93 |
| Axicabtagene Ciloleucel  group | | |
| EASIX at point A | 0.72 | 0.62-0.85 |
| EASIX at point B | 0.72 | 0.61-0.82 |
| m-EASIX at point A | 0.68 | 0.61-0.79 |
| m-EASIX at point B | 0.73 | 0.64-0.87 |

AUC: area under the curve. CI: confidence interval. Corrected by bootstrap based on 100 replicates for each analysis.

Correspondence of time points: A: pre-infusional, B: 24-48h post-infusion. ICU: Intensive Care Unit. No patient in Cesnicabtagene autoleucel group presented ≥grade 3-4 toxicities; therefore, this group was not included in this sub-analysis. The other subgroups of CAR T-cell products were not included in this sub-analysis due to their scarce casuistry.
